# Sensing with Superconducting Point Contacts

**DOI:** 10.3390/s120506049

**Published:** 2012-05-10

**Authors:** Argo Nurbawono, Chun Zhang

**Affiliations:** 1 Department of Physics, National University of Singapore, 2 Science Drive 3, Singapore; E-Mail: argo nurbawono@nus.edu.sg; 2 Department of Chemistry, National University of Singapore, 3 Science Drive 3, Singapore

**Keywords:** point contact spectroscopy, superconductivity, andreev reflections

## Abstract

Superconducting point contacts have been used for measuring magnetic polarizations, identifying magnetic impurities, electronic structures, and even the vibrational modes of small molecules. Due to intrinsically small energy scale in the subgap structures of the supercurrent determined by the size of the superconducting energy gap, superconductors provide ultrahigh sensitivities for high resolution spectroscopies. The so-called Andreev reflection process between normal metal and superconductor carries complex and rich information which can be utilized as powerful sensor when fully exploited. In this review, we would discuss recent experimental and theoretical developments in the supercurrent transport through superconducting point contacts and their relevance to sensing applications, and we would highlight their current issues and potentials. A true utilization of the method based on Andreev reflection analysis opens up possibilities for a new class of ultrasensitive sensors.

## 1. Introduction

Since its first discovery over a hundred years ago [1], superconductors have been utilized for various sensing applications, among others. Superconducting quantum interference devices (SQUIDs) for example, are ubiquitous for ultrasensitive magnetic sensors such as magnetic resonance imaging (MRI) in medical applications, thanks to the Josephson effects [2]. Less common applications are point contact Andreev reflection (PCAR) spectroscopies, which are still fairly limited mainly in laboratory demonstrations and theoretical studies. This is due to non-trivial Andreev physics that is involved in the supercurrent transport through point contacts (PC) which requires more rigorous theoretical treatments in order to decipher the underlying physics and therefore to interpret the experimental results correctly. PC can be fabricated with various methods, for example using a sharp or needle like metallic probe with chemically etched tip, which is then pressed onto another metallic surface using a combination of piezoelectric actuator and differential screw mechanism [3]. A combination of reactive ion etching (RIE) and electron beam machining is also common to produce nanobridges [4], which are basically nanoholes drilled through a thin insulator. Another common technique is micro-controlled break junction (MCBJ) [5], which is basically a metallic nanocontact produced with electron beam machining that can be broken up to produce an atomic gap. This gap can be precisely adjusted using a piezoelectric actuator. The contact sizes range from a few nanometers down to a single atom, and therefore the transport through these PCs is mainly ballistic or under the Sharvin limit [6], where the constriction or the contact size is much smaller than the elastic mean free path of the electrons.

Over the past decade there are mainly two very significant landmarks in the applications of PCAR spectroscopies. The first one is the measurement of magnetic polarization [3,7], which utilizes the fact that Andreev process is suppressed when a supercurrent flows from a superconductor to a magnetic normal metal. The degree of polarization can be precisely measured by fitting the entire differential conductance with an appropriate model based on a semiclassical theory, which would be discussed in detail later in this review. This method has spurred new experimental and theoretical developments in magnetic polarization measurements, partly because the PCAR method is easier and more flexible compared to the older methods such as spin-dependent tunneling planar junctions [8] and spin-resolved photoemissions spectroscopy [9]. The second significant landmark is the experimental determination of individual transmission quantum channels of a superconducting single-atom contact [10–12], utilizing a microscopic Hamiltonian model and nonequilibrium Green's functions technique to fit the current-voltage curves. This was the first time that the details of quantum conduction channels have ever been resolved experimentally after it was first proposed more than fifty years ago by Landauer [13,14]. Since then, the microscopic Hamiltonian theory is becoming the mainstream in the subsequent development of superconducting quantum transport. Many experiments followed after this pioneering work discussing other various aspects such as using different contact materials from niobium [15,16], effects of diffusivity [17], ferromagnetic interface [18], hydrogen adsorption [19], or structural deformation effects [20], *etc.* There are also other more recent exciting experimental developments such as the work of Ji *et al.* [21] and Marchenkov *et al.* [22], and we would also briefly discuss them in the section on experimental surveys.

In order to have a meaningful physical understanding of the PCAR physics, we shall also present a detailed discussions of the theoretical aspects in both semiclassical and quantum pictures. The theoretical discussions in this review shall be divided into two parts. The first part is the summary of the semiclassical treatment based on the famous Blonder–Tinkham–Klapwijk (BTK) theory [23] and its relevant extensions for the PCAR magnetic polarization measurements. The second part is the so-called quantum Hamiltonian theory where we would adopt nonequilibrium Green's function method which is regarded as the most rigorous quantum perturbative technique for dealing with nonequilibrium problems [24]. This formalism fits the atomic point contacts where the conduction consists of only a few quantum channels. We would derive the supercurrent based on the Bardeen–Cooper–Schrieffer (BCS) model of Hamiltonian [25], and highlight some applications of the theory such as to resolve individual quantum channels of a superconducting MCBJ [10], and to study quantum dots coupled to superconducting leads under external radiations [26].

## 2. Experimental Surveys

### 2.1. Magnetic Polarization Measurements

The technique of PCAR spectroscopy has been used for measuring the polarization of ferromagnetic materials [3,7,27], which is mainly driven by the need to search suitable materials for spintronic devices [28,29]. The PCAR method provides easier and more flexible measurements compared to the conventional spin tunneling using planar junctions [8] or spin resolved photoemissions spectroscopy [9]. Unlike the planar junction method, PCAR does not need application of large magnetic field of several Teslas, and there is no constraints in terms of thin film fabrications which impose severe limitations on the types of materials that can be tested. Also, PCAR offers better energy resolutions compared to the photoemission method which is typically limited to ∼1 meV resolutions. The PC and the sample are immersed in a liquid helium bath to keep the temperature below the transition temperature T*_c_*. The positioning and adjustment of the PC employed standard piezoelectric actuators for achieving ideal ballistic contacts. Some cares must be taken to prevent excessive pressure on the tip as this may change electronic properties of the materials and hence the spin polarizations [30]. The current is usually obtained using standard AC lock-in techniques at few kHz frequency.

**Figure 1.**
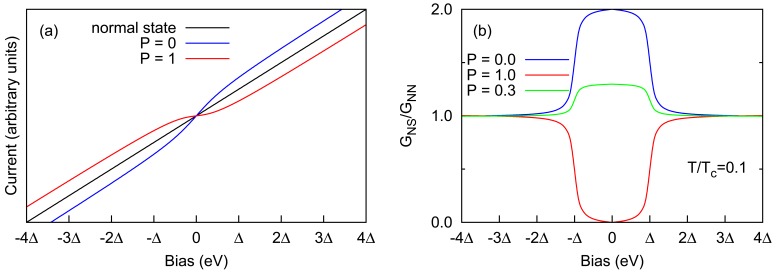
**(a)** typical I-V curves in PCAR measurements. During the normal state (*T* > *T_c_*) the current shows the typical ohmic response. After the PC becomes superconducting (*T* < *T_c_*), non-magnetic systems (*P* = 0) show excess current due to Andreev reflection (AR) process, while ferromagnetic systems (*P* = 1) show suppression of AR process leading to suppression of current; **(b)** Normalized conductance for various polarizations, in the clean metallic limit (*Z* = 0). The bias is in the units of superconducting energy gap.

The PCAR method is based on the fact that the current through the PC differs when the tip is superconducting compared to when it is in normal state. The PCAR method is based on the behaviour of the conductance at very low bias where the current is most dependent on the polarization *P* of the ferromagnet. At low bias electrons enter the gap through Andreev reflection (AR) mechanism, which produces a hole that travels in opposite direction for every electron that enters the gap. The net charge of 2*e* that moves as supercurrent results in the doubling of conductance, *i.e., G*_NS_/*G*_NN_ = 2. This ratio is called the normalized conductance. When the normal metal is a ferromagnet with perfect polarization, *i.e., P* = 1, then the probability for the electron to make a pair with another electron with opposite spin is virtually zero, and therefore AR is completely suppressed at the interface as illustrated in Figure 1(a). This leads to zero conductance, *i.e., G*_NS_/*G*_NN_ = 0. A simple linear interpolation between these two extremes gives, *G*_NS_/*G*_NN_ = 2(1 − *P*), and based on this ballistic assumption, Upadhyay *et al.* [7] and Soulen *et al.* [3,30] independently made the first PCAR magnetic polarization measurements, though the idea for deducing spin polarization from conductance was already proposed by de Jong *et al.* [27]. The theoretical normalized conductance for different polarizations can be seen in Figure 1(b). They fit the entire normalized differential conductance curves for Co, Ni, and some compound ferromagnets as well as Cu. Of course this ballistic assumption is insufficient and the effects of some diffusivity, impurities and surface properties at the contact must be incorporated in order to make better fits to the experimental curves.

Mazin *et al.* [31] and Strijkers *et al.* [32] proposed a straightforward extension to the BTK theory, which then became a more standard method for polarization measurements with PCARs. As the scattering suppresses AR at low bias and creates sharp peaks in the conductance at *eV* = ±Δ, a careful account of the diffusive transport is necessary to obtain more reliable estimate for the polarization measurements. Suppression of AR may be misinterpreted as overestimation of polarization if scattering is not properly accounted for. A different parameterization for the BTK coefficients was then proposed and used to determine the spin polarization measurements in half-metallic CrO_2_ [33]. The modified BTK versions by Mazin and Strijkers are fairly similar and a comparison for CrO_2_ system reveals only 0.02 difference in the polarization measurements, which is about the accuracy of the PCAR method [34]. These details shall be discussed in the theoretical sections. The model also incorporates proximity effects which can reduce the effective gap of superconductors.

Hundreds of related works on PCAR magnetic measurements appeared following these main experimental and theoretical achievements ever since. For instance, Pérez-Willard *et al.* [35] performed PCAR measurements on Al/Co contact fabricated with RIE method [4] and analyzed the dependence of conductance on the temperatures and magnetic fields. The temperature, as predicted by the extended BTK model, reduces the effective superconducting gap and still finds nice agreements with the theory apart from the temperatures close to T*_c_*. Applications of magnetic field parallel to the insulating layer also modifies the Andreev spectra. Magnetic fields reduces the height of the two maxima around the gap and the transition to normal conductance at the threshold field was abrupt. Panguluri *et al.* [36] performed PCAR measurement on MnAs epitaxial films grown on [011] GaAs using Pb and Sn point contacts. They also performed a phonon spectra analysis (*d*^2^*I*/*dV*^2^) of the contacts and concluded that smaller contact diameters are necessary to achieve truly ballistic transport, and to obtain a reliable PCAR measurements contact sizes around 10 nm or smaller are generally preferable. PCAR can also be used to measure spin diffusion lengths. For example Geresdi *et al.* and others [37,38] used PCAR to measure spin relaxation in Pt thin films grown on the top of a ferromagnetic Co layer, where by the temperature dependence was investigated and various sources of the spin relaxation in Pt were identified.

The widespread use of the BTK theory extension for PCAR spectroscopy has been questioned by Xia *et al.* [39] who argued that realistic interface conditions must be considered if PCAR measurements are to be valid after all. From the theoretical works on giant magnetoresistance it is generally known that reflection processes at the interface between nonmagnetic and ferromagnetic materials are strongly spin dependent [40], yet the model used in PCAR experiments never introduced spin-dependent scattering at the interface. Xia *et al.* found that failing to take spin-dependent scattering potential into account would result in poor fitting for Pb/Co systems. Grein *et al.* [41] recently propose spin-active scattering model of PCAR spectra, which include spin filtering and spin mixing effects. They found that the shape of the interface potential has important effects on the spin mixing effects, which probably makes it necessary to reconsider the general validity of some PCAR measurements once again.

### 2.2. Individual Quantum Channel Measurements

The second important landmark in the applications of the PCAR method is to determine the individual transmission coefficients of an atomic point contact (APC) [10,11] or often called quantum point contact (QPC). A typical APC consists only of a small number of eigenchannels and each of them is characterized by a transmission coefficient, *τ_n_*. Each eigenchannel contributes to the conductance by *G*_0_*τ_n_*, where *G*_0_ is the quantum conductance given by *G*_0_ = 2*e*^2^/*h.* The total conductance of an APC is thus given by [13,14],

(1) $$G=\frac{2e^{2}}{h}\sum_{n}\tau_{n}$$

**Figure 2.**
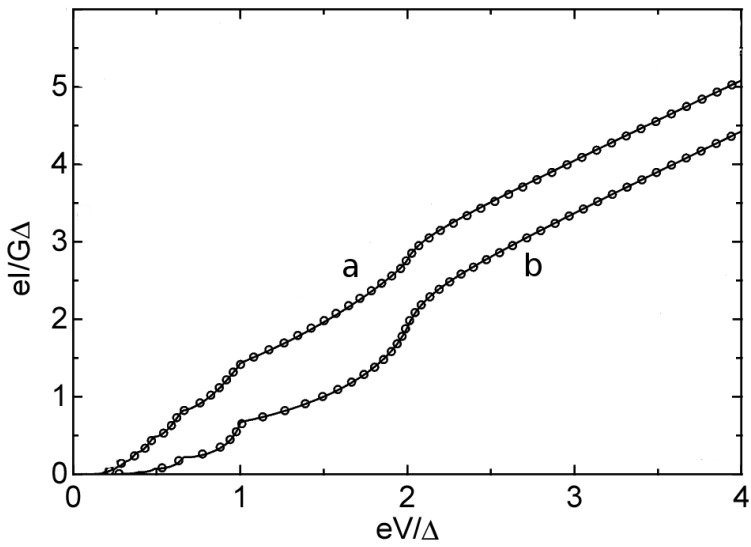
Measured I-V curves for two different Al atomic point contacts having different sets of {*τ_n_*}: *a* = {0.747, 0.168, 0.036} and *b* = {0.519, 0.253, 0.106}. Each *τ_n_* is associated with each valence orbital of Al. The current and voltages are in reduced units, the current is normalized with respect to the total conductance measured by the slope of the I-V at high voltages *eV* > 5Δ. Effectively exact fitting of the experimental data shows the reliability of the theoretical model based on quantum Hamiltonian [11]. Adapted figure reproduced with kind permission from the authors [10]. Other details can be found in the original paper.

Since the transmission coefficient of each channels can take value between zero and unity, the conductance of a single channel is mostly less than *G*_0_ despite the fact that statistically the conductance of an APC tends to be quantized. The quantitative information on individual conductance channels has been inaccessible through normal conductance measurements, but for superconducting systems this can be extracted due to the sensitivity of the so-called sub-gap structures (SGS) of the superconductor at low bias to small changes of each conductance channels. The SGS originates from multiple Andreev reflection (MAR) [42] between two superconductors and the centre normal (vacuum) region, which we shall discuss in detail later in the theory section. This presumably resolves the old question that whether a quantum conductance in the measurements actually corresponds to a number of partially open channels, instead of just one channel. Scheer *et al.* [10] demonstrated using a superconducting Aluminium APC fabricated with MCBJ method, and fitted the time averaged current with the theoretical model based on the quantum Hamiltonian theory [43]. They found that a single Al atomic contact actually corresponds to three partially open eigenchannels, which exactly correspond to the number of the valence orbitals as illustrated in Figure 2. This conclusion is further verified also for Pb and Nb APCs [12]. The study is very fundamental to our understanding in the science of molecular electronics and mesoscopic transport in general. The total current can be analyzed from the independent current contribution of each channels, *i.e.*,

(2) $$I(V)=\sum_{n}I_{n}(V,\tau_{n})$$

(3) $$=\frac{2e}{h}\int_{-\infty }^{\infty }T(E,V)[f_{L}(E)-f_{R}(E)]dE$$

from which the individual *τ_n_* can be deduced, the so-called “PIN code” of the eigenchannels. We shall later discuss the derivation of the transmission terms using quantum Hamiltonian model. Excellent quantitative agreements with the experimental data provide a strong justification for the validity of the subsequent developing theory of superconducting quantum transport.

### 2.3. Magnetic Impurities Measurements

**Figure 3.**
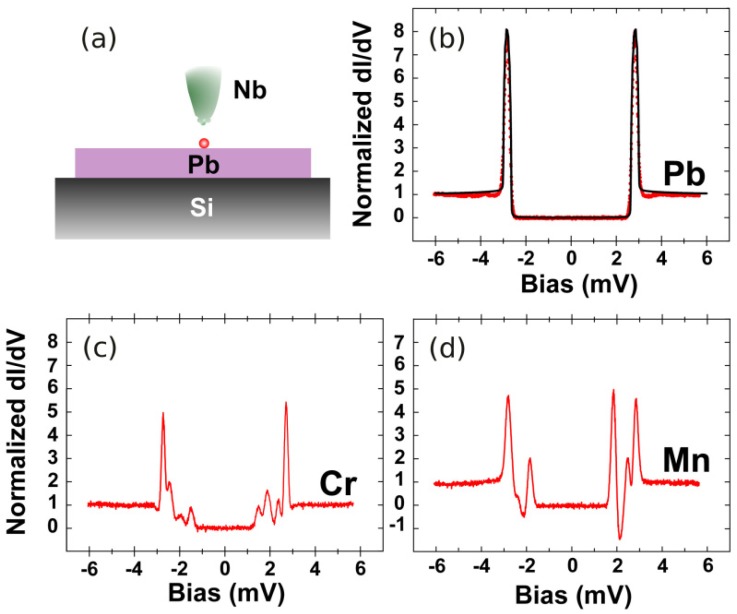
Detecting a single atom magnetic impurities of Mn and Cr on Pb surface with a Nb STM tip. (**a**) is the schematic view of the set up; and (**b**) is the differential conductance (*dI*/*dV*) for a clean Pb surface; (**c**) is for Cr atom where six peaks are detected and (**d**) is for Mn atom where four peaks are detected. The method proposes the use of SGS to identify atomic size magnetic impurities on surfaces. Figures were reproduced and adapted with kind permission from the authors [21].

PCAR spectroscopy has also been used to detect and identify magnetic impurities on superconducting surfaces. Yazdani *et al.* [44] used gold scanning tunneling microscope (STM) tip to study excitations from magnetic adatoms of Mn and Gd on superconducting Nb substrate. Atoms such as Cr, Mn and Gd have been found to reduce the transition temperature T*_c_* of Nb films, and magnetic impurities in general reduce superconducting order parameter and lead to quasiparticle excitations within the superconducting gap [45,46]. Excitations from the magnetic impurities were confirmed by Yazdani *et al.* by comparing them with non-magnetic adatoms such as Ag, which showed almost featureless conductance across the entire bias. Ji *et al.* [21] performed an improved experiment with both the STM tip and the substrate made from superconducting materials Nb and Pb respectively. Unlike Yazdani's work where a quantitative analysis for adatom identifications had been hindered by poor energy resolutions, Ji *et al.* made very significant improvements due to the existence of MAR between the two superconductors which provides high resolution SGS in the conductance, as illustrated in Figure 3. More symmetric SGS structures which are resolved up to 0.1 meV can clearly be seen in the conductance measurements. They claimed that the method can potentially be used to unambiguously detect magnetic adatoms on a superconducting surface, because these spectra are unique fingerprints of the spin states of adatoms, as a result of complex interactions between Andreev bound states (ABS) process and the electronic properties of the adatoms. They also performed similar measurements on dimers of Mn and Cr.

Ji *et al.* used a thin film superconducting Pb which is deposited on a clean Si(111) up to 20 monolayers thick. The superconducting gap of the Pb thin film was found to be 1.30 meV while the Nb STM tip was between 1.44 to 1.52 meV. The effective energy gap of the system turned out to be around 3.0 meV as can be seen in in Figure 3(b) for a clean Pb surface. Different number of peaks with varying intensities were observed for different adatoms. Ji *et al.* suggested that these correspond to each angular momentum channels, though this still requires further investigations. Electron transport process between the STM tip and the adatoms clearly involves only a few quantum channels and the interactions of the ABS with the spin impurities need to be modeled microscopically in order to fit and interpret the experimental data. Apart from the interface issues which are always tricky, first principle calculations of the adatoms combined with suitable model of the superconductors possibly enable unambiguous determination of magnetic adatoms.

### 2.4. Vibrational Mode Measurements

**Figure 4.**
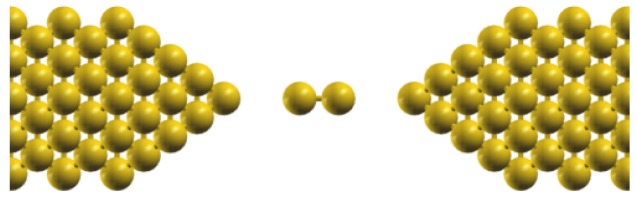
The schematic view of the atomic configurations for measuring vibrational modes of an Nb dimer fabricated with MCBJ technique [22]. The Nb leads were adjusted with piezoelectric movements. The dimer was found to have four modes of vibrations: longitudinal (along the dimer), transverse (up and down), and wagging (torsional) about its centre of mass. These modes affect the MAR tunneling process between the leads and were detected as current singularities inside and outside the superconducting gaps.

Excitations of vibrational modes by traversing electrons have been observed in metallic electrodes attached to nanostructures and molecules such as carbon nanotubes [47,48], hydrogen molecules [49], organic molecules [50,51], gold atomic chains [52], and fullerenes [53]. When a vibrational mode resonates with the bias energy, the conductance can either be enhanced or suppressed by the vibrations. The vibrational energy of the n*^th^* -mode is given by *ħω_n_*, and the bias at which this takes place is *V_n_* = *ħω_n_*/*e*. Thus in such systems, vibrational modes can be detected directly from current measurements alone and to determine the actual modes one must combine it with standard first principle calculations in order to model the complete vibrating molecule. A recent application of PCAR is to study vibrational modes of a suspended Nb dimer conducted by Marchenkov *et al.* [22], as illustrated in Figure 4. The dimer was fabricated with the MCBJ technique, and from previous study based on density functional theory (DFT) calculations and conductance measurements, it was confirmed that the configurations at the tip before the break-up was a Nb dimer, where the symmetry and asymmetry of the dimer position across the gap corresponds to either high or low conductance respectively [54,55]. Though in this particular setup the dimer is made of the same atoms as the leads, the idea is still applicable for other types of molecules to be probed with similar technique. This would enable us to study vibrational modes of a truly isolated molecule, unlike the behaviours of ensembles such as in the conventional IR, UV or NMR spectroscopies [56,57].

The measurements were performed at various temperatures from well below T*_c_* up to 12 K. Resonances for high conductance configurations (the dimer is symmetric between the leads) were analysed which appeared both inside and outside the SGS. Particularly for resonances outside SGS, the so-called over the gap structure (OGS), they observed more symmetric and persistent patterns through out different temperatures until they diminished as *T* > *T_c_*. Unlike the usual SGS which originate from MAR, the OGS do not change positions with bias as the temperature varies. The OGS is not governed by MAR; rather Marchenkov *et al.* suggested that the OGS originated from the atomic scale structural and dynamical properties of the dimer which resonate with the Josephson current oscillations. The exact shapes, amplitudes and widths of these features correspond to different vibronic and electronic coupling regimes. The time dependent electromagnetic fields of the Josephson oscillations resonate with the vibrational eigenmodes of the Nb dimer. Further they compared the frequencies with ab initio calculations based on DFT and found nice agreements for three different modes of vibrations: longitudinal, transverse and wagging. The method offers a new physics to be used to study dynamical properties of small molecules in general.

## 3. Theoretical Surveys

**Figure 5.**
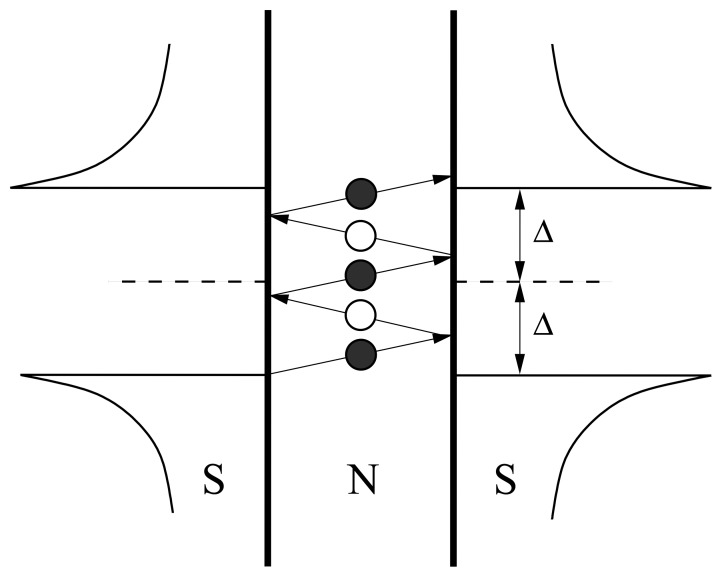
Multiple Andreev reflection (MAR) process in a symmetric superconductor-normal-superconductor (SNS) system with the normal region sufficiently thin to provide ballistic trajectories. The dark particles (electrons) are the antiparticle of the white particles (holes), and the reflection process repeats until they attain sufficient energy to overcome the superconducting gap Δ. The horizontal axes on the superconductor sides represent density of states.

At the heart of the supercurrent transport mechanism is the so-called Andreev reflection (AR) process which can take place when a superconductor is in contact with a normal metal [58]. In the superconductor the quasiparticles form pairs of opposite spins commonly known as the Cooper pairs [59]. For a normal electron to move into the superconductor, it needs to make a pair with another electron with the opposite spin. At bias higher than superconducting gap energy, denoted as Δ, the electron enters as quasielectron which relaxes into the Cooper pair over a charge relaxation distance. At bias *eV* < Δ, superconducting gap prevents direct transfer of single electron states and as a result a hole is reflected back at the interface in order to create a Cooper pair in the superconductor, resulting in the doubling of the conductance as discussed in Section 2.1. When two superconductors are separated by a normal region, a series of electron and hole reflection process take place, which is called multiple Andreev reflections (MAR) [42]. Illustrations can be made with a simple diagram in Figure 5 where a normal region is sandwiched in between two superconductors with identical energy gaps and a small bias *eV* < Δ is applied across the superconductors. The current is oscillating across the junction with a frequency proportional to the bias, *ω* = 2*eV*/*ħ*, known as the AC Josephson frequency, and the MAR process creates SGS in the IV curves.

To illustrate the MAR process, we can use the following arguments: initially an electron from the interface between N and S on the left is accelerated by the external field toward the right, but unable to enter due to the energy gap. This would result in a reflection of a hole moving back to the left. The charge of 2e (one from the electron, the other from the hole moving in opposite direction) increase the supercurrent. The process is repeated until the particle gains sufficient energy to overcome the gap. Octavio *et al.* [42] explains, using the extension of BTK model [23], the SGS in the supercurrent behaviour when the bias is comparable or smaller than Δ. Many researchers have suggested that the SGS are basically current singularities that take place at bias *V* = 2Δ/*en* where *n* = 1, 2, 3, …. However the details of SGS also involve some subtle aspects that are still missing from the semi-empirical approaches, such as the delicate interface properties. An entirely first principle microscopic theory would be needed to quantitatively model the interface natures. A successful quantum theory that can do so would enable PCAR to be used as a reliable sensor with ultrahigh sensitivity, since the SGS provide submili-electronvolt energy resolutions.

### 3.1. The BTK Theory

Now we shall summarize the derivations of the phenomenological treatments for transport through a normal-superconducting (NS) interface of the famous BTK theory [23]. First, let us discuss some elementary results of the Bogoliubov de Gennes equation from which the BTK theory is derived. Readers who are not familiar with superconductivity can consult some well known references [59].

#### 3.1.1. The Bogoliubov de Gennes Equation

The Bogoliubov de Gennes equation [60] describes quasiparticles of electrons and holes in superconductors, analogous to the way Schrödinger equation describes electrons and holes in normal solids. Using the standard two state basis of electron-like and hole-like states, we can describe the wave function as,

(4) $$\psi (x,t)=\left[\begin{array}{c} f(x,t)\\ g(x,t) \end{array}\right]$$

and the Bogoliubov de Gennes equation reads,

(5) $$i\hbar \frac{\partial \psi (x,t)}{\partial t}=\left(\begin{array}{cc} H(x) & \Delta (x)\\ \Delta (x) & -H(x) \end{array}\right)\psi (x,t)$$

where,

(6) $$H(x)=-\frac{\hbar^{2}}{2m}\frac{d^{2}}{dx^{2}}+V(x)-E_{F}$$

**Figure 6.**
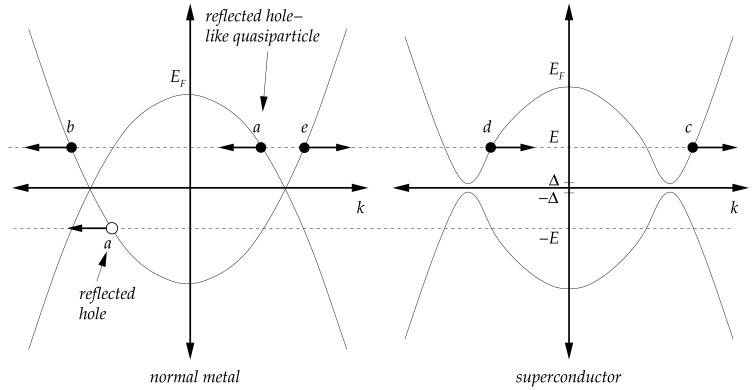
Band diagram for N (left) S (right) interface for the BTK model. The superconducting energy gap in reality is much smaller to Fermi energy (Δ ≪ *E_F_*). Label *e* is the incident electron, *a* is Andreev reflection, *b* is normal reflection, *c* is electron like transmission, and *d* is hole like transmission. Figures are adapted from reference [23].

Δ(*x*) is the spatially dependent superconducting energy gap (or quasiparticle coupling) and *E_F_* is the Fermi energy. The mathematical structure of the equation implies time reversed dynamics of the holes compared to that of the electron quasiparticles. For the simplest scenario where we have Δ(*x*) = Δ and *V*(*x*) = 0, we can have an eigenfunction solution of the form,

(7) $$\psi (x,t)=\left[\begin{array}{c} u\\ v \end{array}\right]\exp^{i(kx-\omega t)}$$

which gives the eigenenergy solution,

(8) $$E^{2}={\left(\frac{\hbar^{2}k^{2}}{2m}-E_{F}\right)}^{2}+\Delta^{2}$$

and the sketch of this energy can be seen in Figure 6 for a normal metal (Δ = 0) and a superconductor (Δ > 0). The positive solution of the energy refers to the electron quasiparticles and the negative one to hole quasiparticles. The superconducting energy gap is introduced whenever Δ > 0, and this is typically in the order of 1 meV for elemental (low T*_c_*) superconductors, while *E_F_* is several eV in magnitude. Another useful quantity is the density of states (DOS) which can be derived from elementary solid state physics,

(9) $$\rho (k)dk=\frac{V}{{\left(2\pi \right)}^{3}}4\pi k^{2}dk$$

and a simple expression for the DOS ratio between the superconducting state to the normal state can be easily derived. Assuming equal Fermi energy between N and S, (*E_F_*)*_N_* = (*E_F_*)*_S_*, and in the limit of small energy range compared to the Fermi energy, we have,

(10) $$\frac{\rho_{S}(E)}{\rho_{N}(E)}=\rho (E)=\frac{E}{\sqrt{E^{2}-\Delta^{2}}}$$

for *E* > Δ and zero otherwise.

#### 3.1.2. Deriving Supercurrent in the BTK Theory

The original BTK theory solves the scattering conditions to obtain reflection and transmission probabilities at the interface between normal metal and superconductor using the simplest possible assumptions. First, BTK theory assumes equal Fermi energy between normal metal and superconductor. Second, the superconducting gap Δ(*x*) is assumed to be spatially independent. In reality, when a superconductor is in contact with a normal metal, there will be some proximity effects [60] due to diffusions of some Cooper pairs into the metal, which reduces the effective gap at the superconsuctor interface. Proximity effects require spatially dependent Δ for a certain length scale around the interface, however in the BTK theory we shall neglect these effects and assume a sudden change of Δ. Third, we shall neglect interactions in both the superconductor and the metal, *i.e., V*(*x*) = 0, for regions deep inside the conductors and in the vicinity of *x* → 0 we can model a simple interface scattering potential such as *V*(*x*) = *Hδ*(*x*) where *H* is the strength of the scattering potential. Such a simple (but unrealistic) scattering potential allows for analytical spatial solutions for the wave function as follows,

(11) $$\psi_{N}(x)=\left[\begin{array}{c} 1\\ 0 \end{array}\right]e^{i(k_{F}+k_{N})x}+a\left[\begin{array}{c} 0\\ 1 \end{array}\right]e^{i(k_{F}-k_{N})x}+b\left[\begin{array}{c} 1\\ 0 \end{array}\right]e^{-i(k_{F}+k_{N})x}$$

(12) $$\psi_{S}(x)=c\left[\begin{array}{c} u\\ v \end{array}\right]e^{i(k_{F}+k_{S})x}+d\left[\begin{array}{c} v\\ u \end{array}\right]e^{i(-k_{F}+k_{S})x}$$

The wave-numbers *k_N_* and *k_S_* are measured from the Fermi-wave number *k_F_*. Referring to Figure 6, the incident electron *e* has probability of unity, and it can experience Andreev reflection (*a*) or normal reflection (*b*) an the interface. The transmission can take in the form of electron-like (*c*) or hole-like (*d*) quasiparticles in the superconductor. Boundary conditions at the interface give,

(13) $$\psi_{N}(0)=\psi_{S}(0)=\psi (0)$$

(14) $$\psi_{S}^{\prime }(0)-\psi_{N}^{\prime }(0)=H\frac{2m}{\hbar^{2}}\psi (0)$$

This allows for the solutions of the coefficients and therefore the probabilities, *A* = |*a*|^2^, *B* = |*b*|^2^, *etc.* The expressions for *A* and *B* are listed in Table 1, while the transmission probabilities *C* and *D* can be calculated from conservation of probability *C* + *D* = 1 − *A* − *B*, but we do not really need their expressions directly in order to derive the current later. The dimensionless quantity *Z* is defined as

$$Z^{2}=\frac{mH^{2}}{2\hbar^{2}E_{F}}$$

often called the barrier strength, representing the strength of the scattering potential *Hδ* (*x*). Now we consider those energies less than the gap energy, *i.e.*, |*E*| < Δ. The incident electrons cannot enter the superconductor as quasiparticles, therefore *A* + *B =* 1. If *Z* = 0, all electrons are Andreev reflected, (*A* = 1, *B =* 0), while for *Z* > 0 some electrons are normally reflected, (*A* < 1, *B* > 0). To resolve this we need to consider normal-normal (NN) interface by letting Δ → 0 or *ρ* → 1. The transmission, evaluated as 1 − (*A* + *B*) is given by,

(15) $$T=\frac{1}{1+Z^{2}}$$

which is the standard result for delta potential scattering, and for Andreev reflection probability at the Fermi energy is given by,

(16) $$A={\left[\frac{1}{1+2Z^{2}}\right]}^{2}$$

which is roughly the square of the normal transmission. This reflects the fact that AR process requires simultaneous transmission of two independent electrons.

After we know the probabilities *A* and *B*, we are ready to calculate the current, which can be deduced either from the left (normal metal) or the right (superconductor) hand side of the interface. Let us consider from the normal metal side: at energy interval *δE*, there is a current contribution to the right from the incident electron, a current contribution from AR which reflects holes to the left, *i.e.*, current to the right, and the normal reflection that contributes current to the left. Summing up all these we have,

(17) δI(E)=−eAv(E)ρ(E)[1+A(E)−B(E)]f(E)δE

where *e* is the electronic charge, 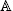 is the point contact cross sectional area, *v*(*E*) is the electron velocity, *ρ*(*E*) is the DOS, and *f*(*E*) is the Fermi-Dirac distribution function. There is also equivalent current flowing to the left from the superconductor, but it has a different Fermi–Dirac distribution function due to the applied bias,

(18) δI(E)=−eAv(E)ρ(E)[1+A(E)−B(E)]f(E−eV)δE

and the total current can be written as,

(19) I=eA∫v(E)ρ(E)[1+A(E)−B(E)][f(E−eV)−f(E)]dE

The integration is mainly over a small energy region around the Fermi level since the term [*f*(*E* − *eV*) − *f*(*E*)] is zero for large energy. In practice, *eV* ∼ Δ ≪ *E_F_*, and thus the velocity and DOS can be taken as constants,

(20) I=eAvρ∫[1+A(E)−B(E)][f(E−eV)−f(E)]dE

The conductance defined as *G* = *dI*/*dV* can be derived for both NN and NS system, giving the conductance ratio of NN to NS as

(21) $$\frac{G_{\text{NS}}}{G_{\text{NN}}}=-(1+Z^{2})\int [1+A(E)-B(E)]f^{\prime }(E-eV)dE$$

which is the main results of the celebrated BTK theory. *f*′(*E*) refers to the derivative of *f*(*E*) with respect to energy. To calculate the current through SNS systems, Octavio *et al.* combined two BTK formulations and used it to explain MAR effects in SNS junctions [42]. Interested readers can refer to the original paper for details.

In order to extend the BTK theory to measure the spin polarizations of ferromagnets, Mazin *et al.* [31] and Strijkers *et al.* [32] proposed that the current *I* is a superposition of a fully polarized current *PI* and a fully non-polarized current (1 − *P*)*I.* The non-polarized current can be calculated using the standard BTK theory while the polarized current needs to be calculated with modified expressions for the reflectivities *Ã* and *B̃*. The modified constants are determined as follows. The fully polarized current consists of one electron spin species only, therefore there is no Andreev reflection, *i.e., Ã* = 0 and *B̃* + *C̃* + *D̃* = 1. At small energies |*E*| < Δ, there is no transmission, implying *B̃* = 1 [32]. For |*E*| < Δ, *B̃* can be determined by assuming that the ratio between normally reflected and transmitted electrons is independent of the polarization, in other words,

(22) $$\frac{B}{C+D}=\frac{\tilde{B}}{\tilde{C}+\tilde{D}}$$

that subsequently gives,

(23) $$\tilde{B}=\frac{B}{1-A}$$

Complete tabulations of *Ã* and *B̃* can be found in the original paper by Strijkers *et al.* [32]. However, Mazin *et al.* proposed a slightly different approach that, for electron with energy above the superconducting gap, describes Andreev reflected holes as spatially decaying evanescent wave with finite probability but carrying no current. This difference turns out to be a minor issue as they differ only by a negligible amount when used to interpret the experiments [34]. The conductance ratio for the spin polarized system is hence given by,

(24) $$\frac{G_{\text{NS}}}{G_{\text{NN}}}=-P(1+Z^{2})\int [1+\tilde{A}-\tilde{B}(E)]f^{\prime }(E-eV)dE-(1-P)(1+Z^{2})\int \left[1+A(E)-B(E)\right]f^{\prime }(E-eV)dE$$

In the metallic limit of perfect contact, there is perfect transparency (*Z* = 0) and the normalized conductance ratio for zero bias is simply given by 2(1 − *P*) as stated earlier in the previous section on the experimental surveys.

### 3.2. Quantum Hamiltonian Theory

In this section we shall summarize a model based on quantum Hamiltonian theory, whose origin can be traced back from the early work by Bardeen who proposed a microscopic Hamiltonian approach for tunneling junction problems [61]. We shall adopt nonequilibrium Green's function (NEGF) formalism to formulate relevant physical quantities. NEGF is a big topic on its own, and readers who are not familiar with its formalism are recommended to browse reference [24], and perhaps some many body topics such as reference [62] and [63]. The historical accounts for the developments of the theory for superconducting resonant tunneling systems can be found in the well known references [43,64–70], and readers who are interested in the details should consult the original papers. In particular, we shall illustrate in detail the method by Sun *et al.* [67] for the supercurrent formulation. The quantum Hamiltonian theory is based on the Bardeen–Cooper–Schrieffer (BCS) model [25], and it still has free adjustable parameters such as the tunneling Hamiltonian and the leads. In order to have a truly first principle method which takes into account the real atomic structure of the device, the theory of superconductivity needs to be combined, for example, with density functional theory (DFT). Fortunately such formalisms are already under developments [71,72] and by combining this formalism with NEGF would enable a first principle calculation for superconducting transport. This is perhaps the future endeavor for the researchers in the field.

#### 3.2.1. Model Hamiltonian and Current Derivation

**Figure 7.**
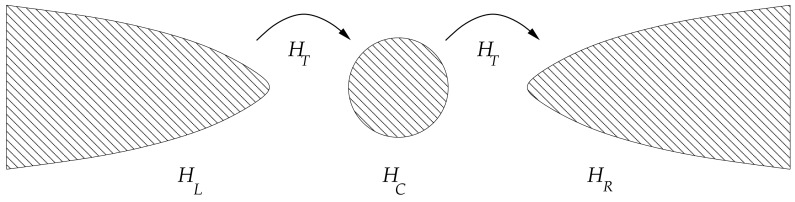
A resonant tunneling system which consists of two superconducting leads and a quantum dot. The system is represented by three subsystem Hamiltonians, *H* = *H_L_* + *H_T_* + *HC* + *H_R_*.

In quantum Hamiltonian theory, a system with two metallic leads can be represented by two independent Hamiltonians, *H_L_* and *H_R_* together with a weak tunneling Hamiltonian between the leads, *H_T_*, that represents coupling by which electrons are transferred from one lead to another. To model experimental systems described in Sections 2.3 and 2.4 where quantum point contacts are used to probe magnetic impurities or molecules, we can add an intermediate centre region where electrons transit before they tunnel to the next lead. This can also be thought of a quantum dot represented by a Hamiltonian *H_C_*. For a vacuum region between the leads such as in Section 2.2 we do not need *H_C_*. The schematics for the system is shown in Figure 7. Expressions for the whole system's Hamiltonian can be written as the following,

(25) $$H(t)=H_{L}+H_{T}(t)+H_{C}+H_{R}$$

where [43],

(26) $$H_{L}+H_{R}=\sum_{k,\sigma ,\alpha =L,R}{ɛ}_{k\alpha \sigma }a_{k\alpha \sigma }^{†}a_{k\alpha \sigma }+\sum_{k,\alpha =L,R}\Delta_{k\alpha }a_{k\alpha ↓}a_{-k\alpha ↑}+\text{H}.\text{c}$$

(27) $$H_{C}=\sum_{i,\sigma }{ɛ}_{i\sigma }c_{i\sigma }^{†}c_{i\sigma }+\text{interaction terms}$$

(28) $$H_{T}(t)=\sum_{k,i,\sigma ,\alpha =L,R}t_{k\alpha i}e^{i(\phi_{\alpha }+2eV_{\alpha }t)}a_{k\alpha \sigma }^{†}c_{i\sigma }+\text{H}.\text{c}.$$

The leads are governed by the mean field BCS theory [59]. Momentum index *k* refers to the leads, and index *i* (or *j*) refers to the quantum dot which contains discrete energy levels *ε_iσ_*. *σ* refers to the spin, *V_α_* is the chemical shift due to bias potential across the junction, and *φ_α_* is the superconducting phase of the leads. Operators *a*^(†)^ annihilate (create) particle on their respective leads, while operators *c*^(†)^ do the same for the quantum dot. The time dependent phase is the consequence of the AC Josephson effects in finite bias, and it is incorporated into the tunneling terms following a gauge transformation suggested by Rogovin *et al.* [73]. For superconducting systems governed by the BCS Hamiltonian, we can construct Green's functions as 2 × 2 Nambu (spinor) space [74] similar to previous construction for Bogoliubov de Gennes, and this is due to the anomalous terms in the potential which contain two operators with opposite spins and momentum. Nambu representation provides consistent and convenient form of Green's function required for the evaluation of equation of motion and perturbation theory. The spinor terms are defined as,

(29) $$\alpha_{k}=\left[\begin{array}{c} a_{k↑}\\ a_{-k↓}^{†} \end{array}\right]\;\text{and}\;\alpha_{k}^{†}=\left[a_{k↑}^{†},a_{-k↓}\right]$$

For example we can calculate the (retarded) free propagator g*^r^* for the mean field BCS model as the following,

(30) $$\begin{array}{cc} {\text{g}}^{r}(k,t,t^{\prime }) & =-i\theta (t-t^{\prime })\langle \left\{\alpha_{k}(t),\alpha_{k}^{†}(t^{\prime })\right\}\rangle \\ & =-i\theta (t-t^{\prime })\left[\begin{array}{cc} \langle \left\{a_{k↑}(t),a_{k↑}^{†}(t^{\prime })\right\}\rangle & \langle \left\{a_{k↑}(t),a_{-k↓}(t^{\prime })\right\}\rangle \\ \langle \left\{a_{-k↓}^{†}(t),a_{k↑}^{†}(t^{\prime })\right\}\rangle & \langle \left\{a_{-k↓}^{†}(t),a_{-k↓}(t^{\prime })\right\}\rangle \end{array}\right] \end{array}$$

Evaluations of this term gives [67,68],

(31) $$\sum_{k}{\text{g}}^{r}(k,t,t^{\prime })=-i\theta (t-t^{\prime })\int dɛ\rho^{N}\beta (ɛ)e^{-iɛ(t-t^{\prime })}\left[\begin{array}{cc} 1 & \Delta /ɛ\\ \Delta /ɛ & 1 \end{array}\right]$$

where *ρ^N^* is normal density of states and *β*(*ε*) is a complex term related to the BCS DOS defined as,

(32) $$\beta (ɛ)=\frac{\left|ɛ\right|}{\sqrt{{ɛ}^{2}-\Delta^{2}}}\theta (|ɛ|-\Delta )+\frac{ɛ}{i\sqrt{\Delta^{2}-{ɛ}^{2}}}\theta (\Delta -|ɛ|)$$

Another useful free propagator is the lesser propagator given by,

(33) $$\sum_{k}{\text{g}}^{<}(k,t,t^{\prime })=i\int dɛ\rho^{N}f(ɛ)\text{Re}[\beta (ɛ)]{\text{e}}^{-iɛ(t-t^{\prime })}\left[\begin{array}{cc} 1 & \Delta /ɛ\\ \Delta /ɛ & 1 \end{array}\right]$$

Time-dependent supercurrent across the junction can be derived from the expectation value of the time derivative of the number operator in any one leads, say the left one for convenience,

(34) I(t)=−e〈N˙L〉=ie〈[NL(t),H(t)]〉=2eRe∑i,kTr{Gi,Lk<(t,t)tLi(t)σZ}

The term ${\text{G}}_{i,Lk}^{<}(t,t)$ is called lesser Green's function, which is defined as,

(35) $${\text{G}}_{j,L,k}^{<}(t,t_{1})=i\left[\begin{array}{cc} \langle a_{Lk↑}^{†}(t_{1})c_{j↑}(t)\rangle & \langle a_{L-k↓}(t_{1})c_{j↑}(t)\rangle \\ \langle a_{Lk↑}^{†}(t_{1})c_{j↑}^{†}(t)\rangle & \langle a_{L-k↓}(t_{1})c_{j↓}^{†}(t)\rangle \end{array}\right]$$

and the term **t***_Li_*(*t*) is tunneling matrix given by,

(36) $${\text{t}}_{Lj}(t)=\left[\begin{array}{cc} t_{Lj}e^{i(\phi_{L}+2eV_{L}t)} & 0\\ 0 & -t_{Lj}e^{i(\phi_{L}+2eV_{L}t)} \end{array}\right]$$

The term *a_z_* is the Pauli matrix,

(37) $$\sigma_{Z}=\left[\begin{array}{cc} 1 & 0\\ 0 & -1 \end{array}\right]$$

The next step is to express the current in terms of the free propagator of the leads and Green's function of the quantum dot. This can be done through NEGF procedure where the corresponding time-ordered Green's function for ${\text{G}}_{i,Lk}^{<}$ is evaluated with NEGF time contour integral, followed by Langreth's analytical continuation. This gives the expression for ${\text{G}}_{i,Lk}^{<}$ as the following,

(38) $${\text{G}}_{j,Lk}^{<}(t,t)=\sum_{i}\int dt^{\prime }\left({\text{G}}_{ji}^{r}(t,t^{\prime }){\text{t}}_{Li}^{*}(t^{\prime }){\text{g}}_{Lk}^{<}(t^{\prime }-t)+{\text{G}}_{ji}^{<}(t,t^{\prime }){\text{t}}_{Li}^{*}(t^{\prime }){\text{g}}_{Lk}^{a}(t^{\prime }-t)\right)$$

where the quantum dot's Green's functions are given by,

(39) $${\text{G}}_{ij}^{r}(t,t_{1})=-i\theta (t-t_{1})\left[\begin{array}{c} \langle \left\{c_{i↑}(t),c_{j↑}^{†}(t_{1})\right\}\rangle \langle \left\{c_{i↑}(t),c_{j↓}(t_{1})\right\}\rangle \\ \langle \left\{c_{i↓}^{†}(t),c_{j↑}^{†}(t_{1})\right\}\rangle \langle \left\{c_{i↓}^{†}(t),c_{j↓}(t_{1})\right\}\rangle \end{array}\right]$$

(40) $${\text{G}}_{ij}^{<}(t,t_{1})=i\left[\begin{array}{c} \langle c_{j↑}^{†}(t_{1})c_{i↑}(t)\rangle \langle c_{j↓}(t_{1})c_{i↑}(t)\rangle \\ \langle c_{j↑}^{†}(t_{1})c_{i↓}^{†}(t)\rangle \langle c_{j↓}(t_{1})c_{i↓}^{†}(t)\rangle \end{array}\right]$$

We can then substitute these into **G***^<^* and write out the current equation. For simplicity in the current example we can include only one localized level in the quantum dot, *i.e.*, transport is only through a single eigenchannel. Using the expressions for the BCS free propagators in the previous chapter and after rearranging the terms we would obtain,

(41) I(t)=−2eIm∫−∞tdt1∫dɛ2πeiɛ(t−t1)Tr{[Re(βL(ɛ))fL(ɛ)Gr(t,t1)+βL∗(ɛ)G<(t,t1)]ΓL∑∼L(ɛ)σz}

and the term Σ̃*_L_*_/_*_R_*(*ε*) is a product term from the rearrangements defined as,

(42) $${\tilde{\sum }}_{L/R}(ɛ)=\left[\begin{array}{cc} e^{ieV_{L/R}(t_{1}-t)} & -\frac{\Delta_{L/R}}{ɛ}e^{-i(\phi_{L/R}+eV_{L/R}(t_{1}+t))}\\ -\frac{\Delta_{L/R}}{ɛ}e^{i(\phi_{L/R}+eV_{L/R}(t_{1}+t))} & e^{-ieV_{L/R}(t_{1}-t)} \end{array}\right]$$

The term Γ*_L_* is the line width matrix function, a product of interlevel tunneling matrices and the normal density of states *ρ^N^*,

(43) $$\Gamma_{L;ij}(t,t_{1})=2\pi {\text{t}}_{Li}(t){\text{t}}_{Lj}^{*}(t_{1})\rho_{L}^{N}$$

which would be a constant in the case of single level quantum dot. Now in order to solve ${\text{G}}_{ij}^{r/<}$ we need to be more specific with the actual form of the interactions in Equation (27) of the quantum dot. For illustrations, we can use the simplest case where the quantum dot is non-interacting, which enables exact evaluations for ${\text{G}}_{ij}^{r/<}$. This corresponds to larger quantum dots where charge screening is sufficiently strong to make the interactions to be accounted only as an overall self-consistent potential. In such simple cases we can use the Dyson and Keldysh equations by first computing the corresponding selfenergies. The selfenergies can be calculated easily from the equation of motions, which take the same form as the resonant tunneling model [66,67],

(44) $$\sum_{L/\text{Rij}}^{r/<}\left(t,t_{1})={\text{t}}_{L/Ri}^{*}(t)\left(\sum_{k}{\text{g}}_{L/Rk}^{r/<}(t,t_{1})\right){\text{t}}_{L/Rj}(t_{1}\right)$$

and using the BCS free propagators stated above we can easily get their explicit forms.

##### 3.2.2. Time Averaged Current and Fourier Transformations

The Josephson current through SNS QPC oscillates at very high frequency, typically in the terahertz range, which makes the time resolved quantities not so easily compared with the experiments. A more convenient way would be to work with the time averaged quantities derived from the Fourier transformation of the correct intrinsic frequencies of the systems. All dynamic quantities can be expanded as harmonics of the fundamental frequency *ω* = 2 *eV, i.e.*,

(45) $$I(t)=\sum_{n}I_{n}e^{\text{in}\omega t}$$

The time average current is derived simply from the zeroth order term *I*_0_. Due to the two-time correlations in the Green's function, we require a transformation that can account them in a consistent manner, and this is done through a so-called double Fourier transform of the Green's functions,

(46) $$G_{mn}(ɛ)=\frac{1}{2\pi }\int_{-T/2}^{T/2}dt_{1}e^{-i(ɛ+n\omega )t_{1}}\int_{-T/2}^{T/2}dte^{-i(ɛ+m\omega )t}G(ɛ,t,t_{1})$$

The retarded Green's function is calculated with the Dyson equation in Fourier transformed form, hence the matrices here are in Fourier space and Nambu space, and for the case of multilevel system it would be the tensor product of all, *i.e.*, [*m, n*] ⊗ [*i, j*] ⊗ [2 × 2] and the retarded function is obtained by straightforward inversion of the whole matrix. The lesser function is calculated with the Keldysh equation and the entire composite matrices are substituted, *i.e.*,

(47) $${\text{G}}^{r}(ɛ)={\left[{\text{g}}^{r}{\left(ɛ\right)}^{-1}-(\sum_{L}^{r}(ɛ)+\sum_{R}^{r}(ɛ))\right]}^{-1}$$

(48) $${\text{G}}^{<}(ɛ)=\left[{\text{G}}^{r}(ɛ)(\sum_{L}^{<}(ɛ)+\sum_{R}^{r}(ɛ)){\text{G}}^{a}(ɛ)\right]$$

The advanced function is obtained from the retarded function by **G***^a^* = [**G***^r^*]^†^, and the time-average current can then be expressed as the zeroth order component of the Fourier transform,

(49) I0=−eπIm∫dɛTr{[fL(ɛ)Re(β(ɛ))G00r(ɛ)+12β∗(ɛ)G00<(ɛ)]ΓL∑∼(ɛ)σz}

**Figure 8.**
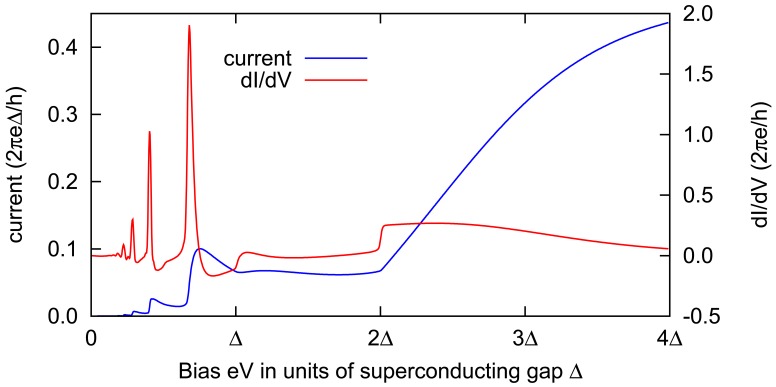
Plot of the time averaged I-V and *dI*/*dV* curves for SNS QPC systems with single level quantum dot (*ε_d_ =* 0). Other parameters are, Γ*_L_* = Γ*_R_* = 0.5Δ and *k_b_T* = 0.1Δ. Rich subgap structures mainly at low bias (*eV* < Δ) can potentially be used to identify the quantum dot's electronic structures and magnetic properties.

The sample plot for the time averaged current and differential conductance (*dI*/*dV*) for single level quantum dot in SNS QPC can be seen in Figure 8. Notice the rich SGS at small bias due to MAR compared to fairly featureless behaviours at higher bias *eV* > 2Δ. The quantum Hamiltonian theory enables us to incorporate more physics into the quantum dot. For example to describe magnetic interactions of the impurities, one may consider a model for *H_C_* of the following,

(50) $$H_{C}=\sum_{i,\sigma }{ɛ}_{i\sigma }c_{i\sigma }^{†}c_{i\sigma }+\sum_{i\neq j}U_{i,j\;}n_{i}n_{j}$$

or other suitable forms of interactions. With this the underlying physics when MAR oscillates across a magnetic impurity can be studied, and general interactions can also be computed with first principle method. For such interacting systems the Green's function may be calculated perturbatively or with other methods. Some examples on such works are by Avishai *et al.* [75] and Pala *et al.* [76]. For a vacuum region between the superconducting leads we do not include *H_C_* and the resulting model is slightly simpler. The model Hamiltonian they used is similar to Equation (25), but in this case without the quantum dot,

(51) $$H(t)=H_{L}+H_{R}+H_{T}(t)$$

where,

(52) $$H_{T}(t)=\sum_{\sigma }\left[te^{i(\phi_{0}+2\text{eVt})}a_{L\sigma }^{†}a_{R\sigma }+t*e^{-i(\phi_{0}+2\text{eVt})}a_{R\sigma }^{†}a_{L\sigma }\right]$$

The tunneling Hamiltonian directly couples left and right leads. For a single eigenchannel system the hopping term *t* is just a constant, and the phase term is the difference between left lead and right lead, *i.e., ϕ*_0_ = *ϕ_L_* − *ϕ_R_* and *eV* = *μ_L_* − *μ_R_*. The equation for the current can then be re-derived using the same procedure as explained in the last sections. Excellent quantitative agreements with the experimental data provide a strong justification for the validity of the microscopic model in the quantum Hamiltonian theory.

##### 3.2.3. Shapiro Effects and External Radiations

Another interesting application of the quantum Hamiltonian theory is for studying the interactions with some external electromagnetic radiations. The frequency range of interests in this case would be in the microwave regions, due to the intrinsic energy scale of typical superconducting energy gaps. The interplay between the AC Josephson effect in superconducting junctions under finite bias with the external radiations exhibit the phenomenon known as the *Shapiro* effects in the supercurrent behaviours [77]. Cuevas *et al.* [78] proposed that the effects from the external radiations of frequency *ω_r_* to some extent can be modeled as effective time dependent voltage, *V_ac_* cos *ω_r_t*, acting on top of the existing AC Josephson frequency. The total effective bias can be written as *V*(*t*) = *V* + *V_ac_* cos *ω_r_t*, and the time dependent phase in the tunneling Hamiltonian becomes

(53) $$\phi (t)=(\phi_{0}+\omega t+\alpha \cos\omega_{r}t$$

where *α* is a measure of the coupling strength with the external radiations. The Fourier series expansion of the current takes the following form,

(54) $$I(t)=\sum_{m,n}{\text{I}}_{n}^{m}\exp\left[i(n\phi_{0}+n\omega t+m\omega_{r}t)\right]$$

For a superconducting QPC system with a featureless barrier, *i.e.*, a vacuum region between two superconducting leads, Cuevas *et al.* managed to compute the supercurrent numerically with the use of Bessel basis functions. They found that the Shapiro effects take place at bias *V* = (*m*/*n*)*ħω_r_*/*2e*, where *m* and *n* are integers. The effects from external radiations are basically current singularities that are distinct from the fundamental SGS of the QPC, since each singularity takes place at infinitely short bias interval and appear as prominent spikes. Chauvin *et al.* [79] have experimentally confirmed this with very good agreements with the model, except for very low bias regions.

For superconducting QPC with a quantum dot at the centre, the localized energy levels at the quantum dot exhibit another intriguing physics upon exposure to external radiations, at least in two ways. First, in semiclassical limit the external field would oscillate the entire set of localized energy levels in unison. Second, absorptions and emissions of the photons would also stimulate interlevel transitions as the electrons tunnel through the quantum dot, and both would affect MAR process inside the quantum dot and hence the supercurrent behaviours. However, in order to do time averaged analysis, one needs to perform multi-frequency Fourier transformation on the dynamical quantities because of the two frequencies dependence of the phase factor. This is non-trivial particularly when the frequencies are non-commensurate, *i.e.*, when their ratio is irrational. To slightly simplify the problem, one may consider replacing one of the superconducting leads with a normal lead (SNN system) and use the gauge where the bias potential at the superconducting lead is zero, thereby eliminating time dependence term from the AC Josephson effects [26].

External radiations can be modeled semiclassically adopting typical dipole approximations [80],

(55) $$H_{C}(t)=\sum_{i,\sigma }\left[{ɛ}_{i}+A\cos(\omega t)]c_{i\sigma }^{†}c_{i\sigma }+\sum_{i\neq j,\sigma }B\cos(\omega t)c_{i\sigma }^{†}c_{j\sigma }\right]$$

**Figure 9.**
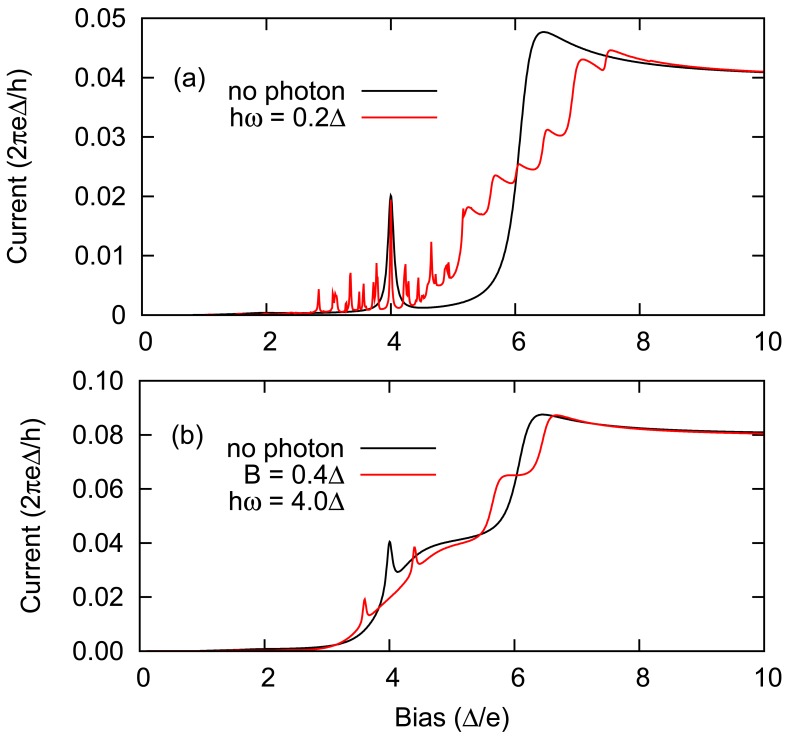
Effects of single mode external radiations on SNN transport in weak coupling limit. (**a**) Time averaged current for a single-level quantum dot in SNN system and the effects of single level oscillations upon external radiations. The external radiations create current resonances at interval *ħω* and preserve the main DC resonance at *eV* = 4Δ; (**b**) Time averaged current for a symmetric two-level quantum dot in SNN system and the effects of interlevel transitions due to the external radiations. In this case the external radiations can only affect the transport when the frequency is equal to the energy difference between the localized levels, *i.e.*, at Rabi frequency *ħω* = (*ε*_1_ − *ε*_2_). The main DC resonance at 4Δ splits into two, and the separation between the split is equal to 2*B*. The simple relationship provides a way to directly measure the interlevel coupling strength from a simple current measurements [26].

In this case, the Green's function of the quantum dot may be computed with the use of Floquet basis [81], which was found to enable flexible modeling of quantum transitions in a multilevel quantum dot [26]. One can study the effects of localized level oscillations by letting *B* = 0, and it was found that series of resonances appear due to the oscillations and the energy spacing between these resonances is equivalent to the radiation energy as can be seen in Figure 9. On the other hand the effects from interlevel transitions can be studied by simply letting *A* = 0 and transitions was found to produce splitting on the primary DC resonance when radiation frequency is at Rabi frequency. Furthermore, the splits were separated by the energy proportional to the interlevel hopping constant *B*. This provides the possibility for experimental inference of the interlevel coupling strength from simple current measurements. In addition, the details of the quantum dot can greatly affect the transport behaviours such as the symmetry of the quantum dot with respect to the leads [82], the relative energy difference between the localized level and the superconducting gap, electronic interactions *etc.* [83]. If these additional factors are not carefully taken into account, any physical deductions based on the incomplete model would potentially lead to false conclusions.

## 4. Conclusions

Intrinsically small energy gap in superconducting PCAR spectroscopy provides a promising candidate for ultrasensitive sensors, making use of the AR process which carries rich physics at the contacts. AR process in NS systems can be used to probe spin polarizations of ferromagnetic materials with convenience and high precision compared to the conventional methods. Theoretical developments in this area are mainly based on the BTK theory, which had begun earlier and has become a relatively mature theory to be used in spin polarization measurements. However, some problems still remain that relate to various delicate details of the surface properties at the contacts which have been treated phenomenologically.

Atomic contacts such as STM tips and MCBJ have discrete eigenchannels and the quantum Hamiltonian theory combined with NEGF enables rigorous descriptions of the complex transport properties of MAR. The method also has promising potentials to be extended for a fully first principle method if we combine the existing first principle superconductivity theory [71,72] with NEGF, which is a possible future research direction for anyone working in this field.

## Figures and Tables

**Table 1.** Table for coefficients A (Andreev reflection) and B (normal reflection).

***E* < Δ**	***E* < Δ**
*A* = Δ^2^/[*E*^2^ + (Δ^2^ − *E*^2^)(1 + 2*Z*^2^)^2^]	*A* = (*ρ*^2^ − 1)/[*ρ*+(1+2*Z*^2^)]^2^
*B* = 1 − *A*	*B* = 4*Z*^2^(1 + *Z*^2^)/[*ρ*+(1+2*Z*^2^)]^2^]
